# Proteostasis Disruption by Proteasome Inhibitor MG132 and Propolin G Induces ER Stress‐ and Autophagy‐Mediated Apoptosis in Breast Cancer

**DOI:** 10.1002/fsn3.70632

**Published:** 2025-07-13

**Authors:** Jih‐Tung Pai, Lei‐Po Chen, Hsuan‐Jui Chang, Shih‐Wei Wang, Yann‐Lii Leu, Cheng‐Ta Lai, Meng‐Shih Weng

**Affiliations:** ^1^ Division of Hematology and Oncology Tao‐Yuan General Hospital, Ministry of Health and Welfare Taoyuan City Taiwan; ^2^ Department of Medicine MacKay Medical College New Taipei City Taiwan; ^3^ Division of Spine Surgery, Department of Orthopedic Surgery MacKay Memorial Hospital New Taipei City Taiwan; ^4^ Department of Nutritional Science Fu Jen Catholic University New Taipei City Taiwan; ^5^ Institute of Biomedical Sciences MacKay Medical College New Taipei City Taiwan; ^6^ Graduate Institute of Natural Products, College of Pharmacy Kaohsiung Medical University Kaohsiung City Taiwan; ^7^ Graduate Institute of Natural Products, College of Medicine Chang Gung University Taoyuan City Taiwan; ^8^ BioBank Chang Gung Memorial Hospital at Linkou Taoyuan City Taiwan; ^9^ Division of Colon and Rectal Surgery, Department of Surgery MacKay Memorial Hospital Taipei Taiwan

**Keywords:** autophagy, propolin G, proteasome inhibitor, protein homeostasis

## Abstract

The maintenance of protein homeostasis, commonly referred to as proteostasis, is critical for the proper functioning of cells. Disruptions in protein degradation pathways can result in proteotoxic stress, which may ultimately lead to cellular apoptosis. Targeting the dysregulation of proteostasis has emerged as a promising approach in cancer therapy. Propolin G, a c‐prenylflavanone derived from Taiwanese propolis, has demonstrated anticancer properties; however, its underlying mechanisms remain largely unexplored. In this study, we evaluated the combined effect of propolin G and the proteasome inhibitor MG132 on breast cancer cells. While individual treatments with MG132 (1 μM) or propolin G (10 μM) exhibited minimal effects on cell viability, their combination resulted in a synergistic suppression of proliferation and induction of apoptosis, as indicated by a combination index (CI) of 0.63. This combined treatment significantly reduced proteasome activity, leading to the accumulation of polyubiquitinated proteins. Mechanistically, apoptosis was mediated through the activation of the PERK/ATF4/CHOP signaling pathway and autophagy, as evidenced by increased expression levels of ULK1, Beclin1, ATG5, and LC3‐II. These findings highlight the potential of targeting proteostasis disruption as an effective anticancer strategy in breast cancer. The combination of propolin G and MG132 may leverage cancer‐specific vulnerabilities and possess translational potential for anticancer therapy.

## Graphical abstract

- Combination treatment with MG132 and propolin G, a c‐prenylflavanone from Taiwanese propolis, synergistically suppressed proteasome activity, leading to proteotoxic stress‐mediated apoptosis in breast cancer.
- This combination treatment triggers apoptosis by activating the PERK‐mediated unfolded protein response (UPR) pathway.
- Autophagy‐mediated cell death is a key mechanism contributing to apoptosis induction by this combination treatment.

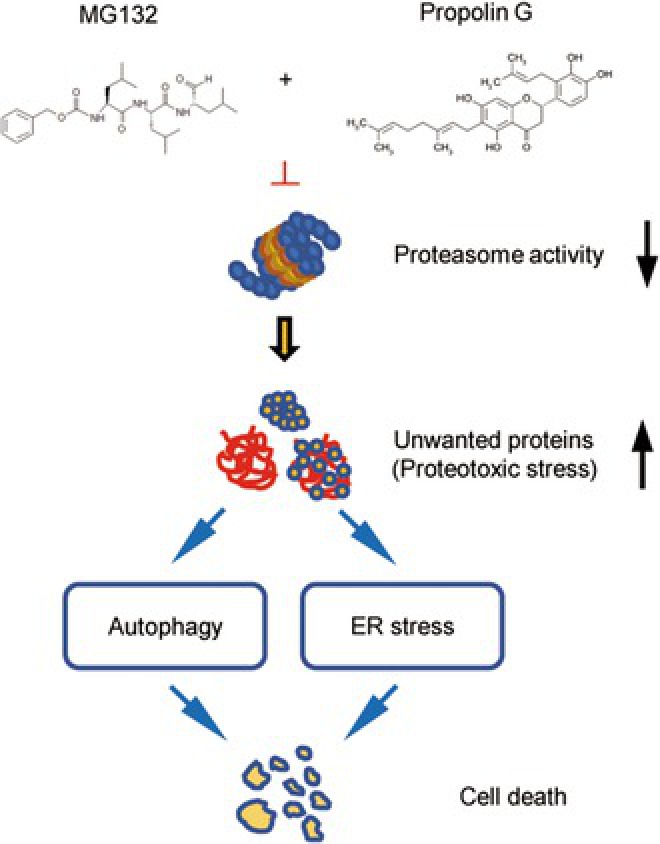

## 1 Introduction

Cancer cells exhibit a significantly higher rate of division compared to normal cells, facilitating their rapid growth and proliferation. These cells aberrantly activate transcriptional and translational mechanisms to synthesize substantial quantities of proteins that are essential for cell division, survival, and various cellular functions (Ruggero 2013). However, the elevated rates of protein synthesis lead to the accumulation of damaged, immature, unfolded, and misfolded proteins. Consequently, protein degradation systems are crucial for maintaining proteostasis by preventing the accumulation of these proteins within the cellular environment. Cells possess two primary protein degradation systems: the ubiquitin‐proteasome system and the autophagy‐lysosomal system. The proteasome consists of a 19S regulatory subunit and a 20S catalytic core subunit. Within the ubiquitin‐proteasome system, proteins are initially tagged with polyubiquitin. This ubiquitylation process is facilitated by three key enzymes: the E1 ubiquitin‐activating enzyme, the E2 ubiquitin‐conjugation enzyme, and the E3 ubiquitin‐ligase enzyme (Nandi et al. 2006). The polyubiquitinated proteins are recognized by the 19S regulatory subunit, which subsequently undergoes deubiquitination. Following this modification, the proteins are translocated to the 20S catalytic core subunit for degradation (Glickman and Ciechanover 2002). Importantly, the disruption of proteasome activity results in the accumulation of ubiquitinated proteins, leading to proteotoxic stress and ultimately precipitating cell death (D'Arcy et al. 2015; Mir et al. 2023; Tian et al. 2014).

The autophagy‐lysosomal system is a critical protein degradation pathway essential for maintaining proteostasis. Currently, three distinct forms of autophagy have been characterized: namely macroautophagy (hereafter referred to simply as autophagy), microautophagy, and chaperone‐mediated autophagy (CMA). Despite their morphological differences, all forms of autophagy facilitate the delivery of cargo proteins to the lysosome for subsequent degradation (Yang and Klionsky 2010). The autophagy process is orchestrated by autophagy‐related (ATG) proteins, which coordinate the formation of an autophagosome that engulfs cargo proteins. Subsequently, the autophagosome fuses with lysosomes, where the enclosed materials are degraded by lysosomal hydrolases (Nishimura and Tooze 2020). In response to various extra‐ and intracellular stresses, such as starvation, hypoxia, and endoplasmic reticulum (ER) stress, the AMP‐activated protein kinase (AMPK)/mTOR signaling pathway is activated, promoting the assembly of the ULK1 complex (He and Klionsky 2009). Once the ULK1 complex is established, the membrane is isolated from the ER membrane and extends to form an autophagosome. During this membrane extension, the ATG5‐ATG12 complex and the ATG8/LC3 system cooperate to facilitate the membrane's extension until the autophagosome is fully formed (Brier et al. 2019; Zhou et al. 2022). While the induction of autophagy has been shown to exhibit tumor‐suppressive properties, its potential oncogenic role remains under investigation (Amaravadi et al. 2016; Levy et al. 2017). This dual functionality of autophagy is contingent upon various factors, including the type of cancer, the stage of the tumor, specific oncogenic mutations, and the contextual stressors involved.

The suppression of the protein degradation system leads to the accumulation of misfolded proteins, which subsequently induce cell death mediated by ER stress (Obeng et al. 2006; Walter and Ron 2011). To mitigate the damage caused by ER stress, three branches of the unfolded protein response (UPR) are activated: activating transcription factor 6 (ATF6), the inositol‐requiring enzyme 1 (IRE1), and the protein kinase RNA‐like ER kinase (PERK) signaling pathways. The activation of the PERK and IRE1 signaling pathways reduces overall protein synthesis and restricts the influx of proteins entering the ER. Concurrently, the activation of the ATF6 pathway enhances the protein‐folding capacity of the ER (Walter and Ron 2011). However, prolonged activation of the PERK/ATF4 pathway can lead to apoptosis mediated by the transcription factor C/EBP homologous protein (CHOP) during an extended period of ER stress (Mimura et al. 2012; Rozpedek et al. 2016; Walter and Ron 2011). Consequently, the induction of proteotoxicity through the combination of proteasome inhibitors and UPR activators may represent a viable strategy for cancer prevention and treatment.

Breast cancer represents the most prevalent malignancy among women and is the leading cause of cancer‐related mortality in females in Western countries (DeSantis et al. 2014; Zielonke et al. 2021). The rising incidence of breast cancer has necessitated extensive research into its biological characteristics and therapeutic approaches. This malignancy is typically categorized into four primary molecular subtypes: Luminal A, Luminal B, HER2‐overexpressed, and Basal‐like (Prat et al. 2011). Variations in hormone receptor expression among these subtypes result in differing treatment strategies and outcomes. Hormone therapy is frequently utilized for the Luminal A and B subtypes, whereas anti‐HER2 monoclonal antibody therapy is considered the standard of care for HER2‐overexpressed breast cancer (Reinert and Barrios 2015; Wuerstlein and Harbeck 2017). The standard treatment for the Basal‐like subtype, commonly referred to as triple‐negative breast cancer (TNBC), primarily involves chemotherapy or a combination of chemotherapy and monoclonal antibodies targeting vascular endothelial growth factor (VEGF) (Berrada et al. 2010). While Luminal A, Luminal B, and HER2‐positive subtypes of breast cancer typically exhibit favorable treatment outcomes, significant challenges persist concerning mortality rates, recurrence, and treatment‐related adverse effects, particularly in TNBC, which is characterized by limited therapeutic options and poorer prognoses. Consequently, there is an urgent need to identify novel strategies for the treatment and prevention of breast cancer. Recent research has underscored innovative approaches that leverage the induction of proteotoxicity as a potential therapeutic strategy for breast cancer. For example, the co‐inhibition of proteasome subunits β2 and β5 disrupts the synthesis of new proteasome, thereby enhancing the sensitivity of TNBC to cell death induced by proteasome inhibitors (Weyburne et al. 2017). Furthermore, the combined administration of the proteasome inhibitor MG132 and paclitaxel has been shown to inhibit the proliferation of breast cancer cells significantly. The integration of proteasome inhibitors with other clinical agents has demonstrated synergistic anti‐proliferative effects in TNBC cells (Hernandez‐Vargas et al. 2007; Shi et al. 2018; Wen et al. 2023). These findings highlight the potential of combination therapies that incorporate proteasome inhibitors alongside other anticancer agents as a promising strategy for the treatment and prevention of breast cancer.

Propolis has been utilized as a traditional medicine for over a century and has been demonstrated to exhibit a range of biological activities. The biological constituents of propolis are classified according to the species of plant sources, seasonal variations, and geographic distribution (Bankova 2005; Bankova et al. 2014). Among these constituents, c‐prenylflavanones represent a specific category that has been identified in Taiwanese and Okinawan propolis (Chen et al. 2003; Kumazawa et al. 2004; Kumazawa et al. 2008; Popova et al. 2010). Eight c‐prenylflavanones, designated as propolin A through H, have been characterized in Taiwanese propolis. Investigations into the anticancer properties of propolins have revealed mechanisms that promote cell cycle arrest and apoptosis in various cancer cells (Chen, Weng, et al. 2004; Chen, Wu, and Lin 2004; Chen et al. 2007; Huang et al. 2007; Weng et al. 2007). Additionally, propolin C and G have been shown to inhibit cell migration and invasion by disrupting the process of epithelial‐mesenchymal transition (EMT) in lung cancer and TNBC cells, respectively (Pai et al. 2018, 2022). These findings indicate that propolins may serve as potential agents for cancer prevention. Consequently, further exploration of the anticancer effects of propolins in the context of breast cancer treatment and prevention is warranted. In the present study, we assessed the anticancer effects of the combination treatment involving the proteasome inhibitor MG132 and propolin G in breast cancer cells. The results indicated that the combination of MG132 and propolin G produced more significant anti‐proliferative effects through the induction of apoptosis. A substantial reduction in proteasome activity was observed in the group receiving the combined treatment of MG132 and propolin G. Moreover, the activation of the PERK/ATF4/CHOP‐mediated UPR and the induction of autophagy through proteotoxic stress may elucidate the mechanism by which the combination treatment of MG132 and propolin G inhibited cell viability in breast cancer cells. Therefore, the co‐administration of the proteasome inhibitor with propolin G may represent a promising therapeutic strategy for the treatment and prevention of breast cancer.

## 2 Materials and Methods

### 2.1 Reagents and Chemicals

Propolin G was purified by Prof. Yann‐Lii Leu (Chang Gung University), utilizing the methodologies outlined by Pai et al. (2022). The compound 3‐(4,5‐dimethylthiazol‐2‐yl)‐2,5‐diphenyl tetrazolium bromide (MTT) was purchased from BIONOVAS Biotechnology Co. (Toronto, ON, Canada). Additionally, MG132, GSK2606414, STF083010, and chloroquine were obtained from MedChemExpress (Taipei, Taiwan). The proteasome activity assay kit was acquired from Abcam Inc. (Cambridge, UK). Antibodies against cleaved caspase 9, Bcl‐2, Bax, cytochrome c, phosphorylated eIF2α, and vinculin were purchased from Cell Signaling Technology (Beverly, MA, USA). Anti‐ubiquitin and anti‐β‐actin antibodies were acquired from Santa Cruz Biotechnology (Santa Cruz, CA, USA). Furthermore, antibodies against cleaved caspase 3, ULK1, Beclin1, ATG5, LC3‐II, GRP78, PERK, phosphorylated PERK, ATF4, ATF6, CHOP, IRE1, phosphorylated IRE1, and XBP1 were sourced from BioAb Inc. (Taipei, Taiwan).

### 2.2 Cell Culture and Cell Viability Analysis

Breast cancer cell lines MCF7, BT474, MDA‐MB‐231, MDA‐MB‐468, and Hs578T were generously provided by Prof. Yuan‐Soon Ho (China Medical University). The MDA‐MB‐231, MDA‐MB‐468, and Hs578T cell lines were maintained in DMEM‐F12 medium (Hyclone Laboratories, Logan, UT, USA). In contrast, BT474 cells were cultured in Hybri‐Care Medium (Thermo Fisher Scientific Inc., Taipei, Taiwan), while MCF7 cells were incubated in RPMI‐1640 medium (Hyclone Laboratories, Logan, UT, USA). All cultures were supplemented with 10% fetal bovine serum, and the cells were incubated in a 5% CO_2_ atmosphere at 37°C.

For the analysis of cell viability, cells (1 × 10^4^ cells/well) were cultured in 96‐well plates and subsequently treated with MG132 (1, 2.5, 5 μM), propolin G (5, 10, 20 μM), or a combination of MG132 and propolin G for 24 h. Following the treatment, an MTT assay was conducted to assess cell viability. The combination index (CI) value was calculated using CompuSyn software.

### 2.3 Colony Formation Assay

MDA‐MB‐468 cells were seeded (2.5 × 10^3^ cells/well) on 6‐well plates in triplicate and incubated for 24 h. Following this incubation, the cells were treated with MG132 (1 μM), propolin G (10 μM), and a combination of MG132 and propolin G for an additional 24 h. Subsequently, the complete culture media were replaced every 3 days over a period of 14 days. The cells were then washed and fixed with 70% ice‐cold methanol for 30 min. The fixed colonies were stained with 0.1% crystal violet, washed to eliminate dye, photographed, and subsequently quantitated using the Quantity One System (Bio‐Rad Laboratories, Hercules, CA, USA).

### 2.4 Apoptosis Analysis

MDA‐MB‐468 cells (6 × 10^5^ cells/dish) were cultured and subjected to treatment with MG132 (1 μM), propolin G (10 μM), and a combination of MG132 and propolin G for 24 h. Following treatment, the cells were collected and stained with propidium iodide (Sigma Chemical, St. Louis, MO, USA) to analyze the DNA content using a FACScan laser flow cytometer. The sub‐G1 population of cells was subsequently analyzed utilizing Kaluza analysis software (Beckman Coulter, Fullerton, CA, USA).

### 2.5 Proteasome Activity Assay

MDA‐MB‐468 cells (3 × 10^5^ cells/dish) were cultured and subsequently treated with MG132 (1 μM), propolin G (10 μM), and a combination of both for 24 h. Following the treatment, the cells were washed twice with phosphate‐buffered saline (PBS) and then trypsinized using trypsin–EDTA. The resulting cell suspensions were collected and centrifuged at 13,000 rpm for 5 min at 4°C. The cell pellet was lysed by 0.5% NP‐40 and subjected to a second centrifugation at 13,000 rpm for 15 min at 4°C. The supernatant was subsequently collected for the proteasome activity assay.

To assess proteasome activity, the supernatant was incubated with the substrate Succ‐LLVY‐AMC for 30 min at 37°C, in accordance with the manufacturer's instructions. Fluorescence was quantified using an ELISA reader at excitation and emission wavelengths of 350 nm and 440 nm, respectively, resulting in a measurement designated as T1. Subsequently, the plate was re‐incubated at 37°C for an additional 30 min, after which fluorescence was measured again to obtain a second measurement, T2. Proteasome activity was calculated using the formula specified in the protocol.

### 2.6 Western Blot Analysis

Following treatment, cell lysates were extracted using RIPA buffer, and total protein concentration was quantitated. Equal amounts of protein (50 μg/mL) were subjected to sodium dodecyl sulfate–polyacrylamide gel electrophoresis (SDS‐PAGE). Subsequently, the proteins were transferred to polyvinylidene fluoride (PVDF) membranes (Millipore Co., Billerica, MA, USA) and incubated with 5% blocking solution for 1 h. The primary antibodies were then incubated at room temperature for 2 h. After washing the membranes three times with ice‐cold phosphate‐buffered saline containing Tween 20 (PBST), secondary antibodies were applied for further reaction. Chemiluminescence signals were detected using an enhanced chemiluminescence (ECL) solution (Amersham Pharmacia Biotech, IL, USA), and images were captured with a UVP ChemiDoc‐It2 810 Imager (UVP LLC, CA, USA). The expression levels of β‐actin or vinculin were utilized as the internal control.

### 2.7 Statistical Analyses

The results are presented as the mean ± standard deviation (SD) derived from a minimum of three independent experiments. Significant differences among groups were assessed using one‐way ANOVA, followed by appropriate post hoc tests. A *p*‐value of less than 0.05 was considered statistically significant.

## 3 Results

### 3.1 Combination Treatment of Proteasome Inhibitor and Propolin G Enhanced the Cell Cytotoxicity

**FIGURE 1:**
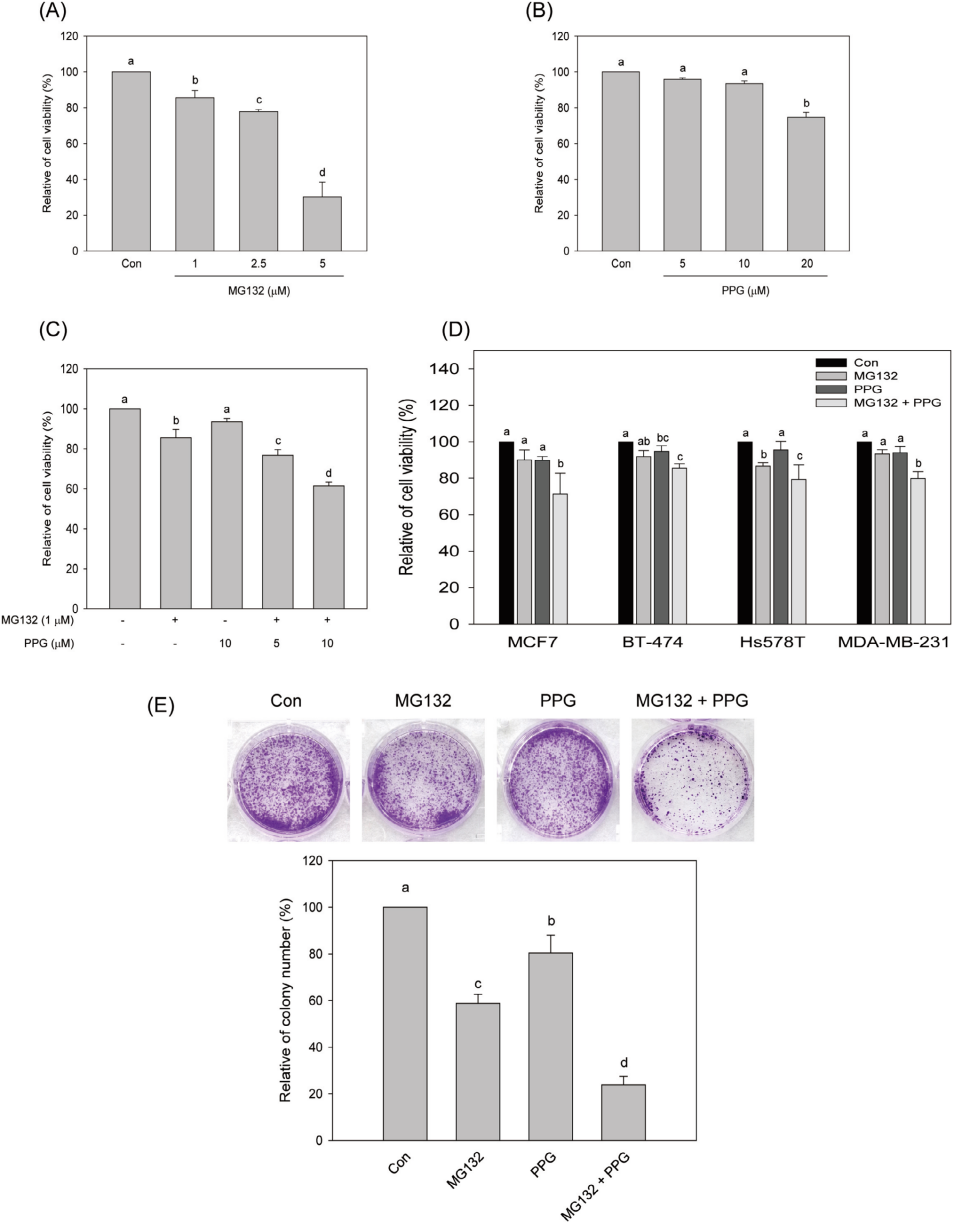
Cytotoxic effects of the combined treatment of MG132 and propolin G on breast cancer cells. MDA‐MB‐468 cells were cultured, and the specified concentrations of (A) MG132, (B) propolin G (PPG), and (C) the combined treatment were administered for 24 h. (D) Five distinct breast cancer cell lines were subjected to treatment with 1 μM of MG132, 10 μM of PPG, and the combination of MG132 (1 μM) and PPG (10 μM) for 24 h. Subsequently, the cytotoxic effects were assessed using the MTT method. (E) MDA‐MB‐468 cells were treated with 1 μM of MG132, 10 μM of PPG, and the combination for 24 h. Following this treatment, the cells were replenished with fresh media and incubated for an additional two weeks. Colony‐forming cells were stained with crystal violet and quantified. The relative colony number was normalized to the control. All results were replicated three times and are presented as means ± SD. Mean values denoted with different subscript letters were found to be significantly different from those of the control (*p* < 0.05).

To assess the cytotoxic effects of the proteasome inhibitor MG132 and propolin G, both individually and in combination, MTT analysis was conducted as outlined in the Materials and Methods section. As demonstrated in Figure 1A, treatment with MG132 for 24 h resulted in a dose‐dependent increase in cytotoxicity in MDA‐MB‐468 cells. Conversely, propolin G alone exhibited significant cytotoxicity only at a concentration of 20 μM (Figure 1B). To evaluate potential synergistic effects, subtoxic or minimally cytotoxic concentrations of MG132 and propolin G were selected for combination treatment. Figure 1C illustrates that co‐treatment significantly enhanced cytotoxicity compared to either agent administered alone. Notably, the combination of 1 μM MG132 and 10 μM propolin G produced the most pronounced cytotoxic response, with a calculated combination index (CI) of 0.63, indicating a synergistic interaction. This combination was subsequently selected for further experimentation.

The cytotoxic effects of the combination treatment were further evaluated in various breast cancer cell lines. As illustrated in Figure 1D, the combination treatment exhibited significant cytotoxicity across all tested cell lines. Notably, MDA‐MB‐468 cells demonstrated greater susceptibility to the combination treatment compared to the other cell lines, as depicted in Figure 1C,D. To assess the long‐term cytotoxic effects, a colony formation assay was conducted. The results indicated that the combination treatment was more effective in inhibiting colony‐forming ability than either MG132 or propolin G administered alone, as shown in Figure 1E.

### 3.2 Combination Treatment of Proteasome Inhibitor and Propolin G Induced Apoptosis in MDA‐MB‐468 Cells

**FIGURE 2:**
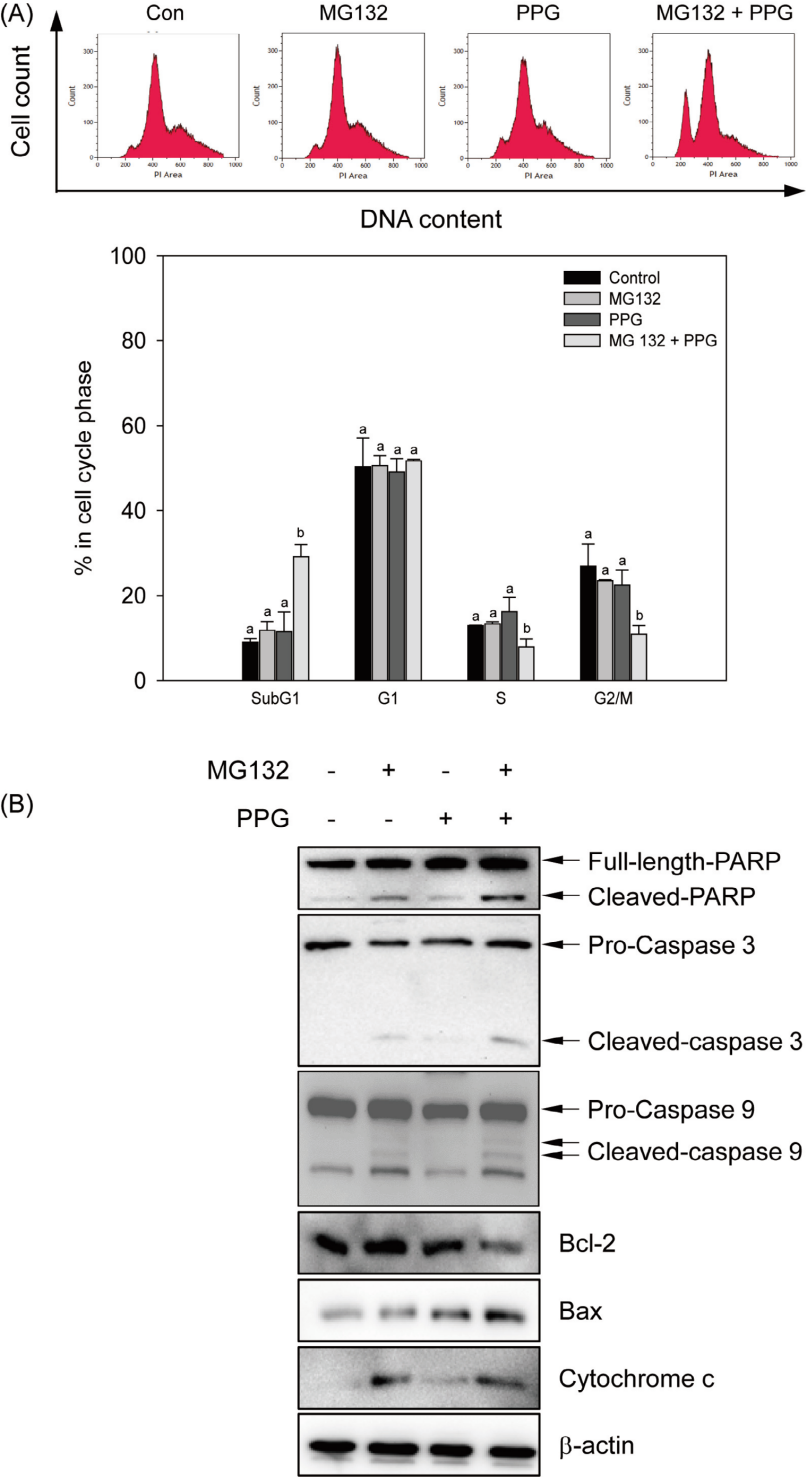
Induction of apoptosis by the combined treatment of MG132 and propolin G on MDA‐MB‐468 cells. MDA‐MB‐468 cells were subjected to treatment with 1 μM of MG132, 10 μM of propolin G (PPG), and the combination of MG132 (1 μM) and PPG (10 μM) for 24 h. (A) Following treatment, cells were collected and stained to assess the apoptotic population using flow cytometry. All experiments were conducted in triplicate, and results are presented as means ± SD. Mean values denoted with different subscript letters were found to be significantly different from those of the control group (*p* < 0.05). (B) Following treatment, cell lysates were extracted, and the expression levels of apoptotic‐related proteins were assessed using western blot analysis.

To further elucidate the molecular mechanisms underlying the cytotoxicity induced by the combination treatment in breast cancer cells, flow cytometry was employed to assess cell death. The sub‐G1 population of cells exhibited an increase from 9.1% ± 0.8% to 29.1% ± 2.8%, indicating a significant rise of approximately 20% as a consequence of the combination treatment. Meanwhile, the S and G2/M populations demonstrated decreases of about 5% and 16%, respectively (Figure 2A). Furthermore, the expression levels of apoptosis‐regulated proteins were examined. The expression of cleaved PARP was significantly elevated in the combination treatment group compared to either MG132 or propolin G treatment alone. Correspondingly, the cleaved forms of caspase‐3 and caspase‐9 were found to be associated with the increase in cleaved PARP. Additionally, the combination treatment resulted in downregulation of Bcl‐2 protein expression while simultaneously upregulating the levels of Bax and cytochrome c proteins (Figure 2B). These findings suggest that the cytotoxic effect of the combination treatment was mediated through the induction of apoptosis in MDA‐MB‐468 cells.

### 3.3 Combination Treatment of Proteasome Inhibitor and Propolin G Suppressed Proteasome Activity in MDA‐MB‐468 Cells

**FIGURE 3:**
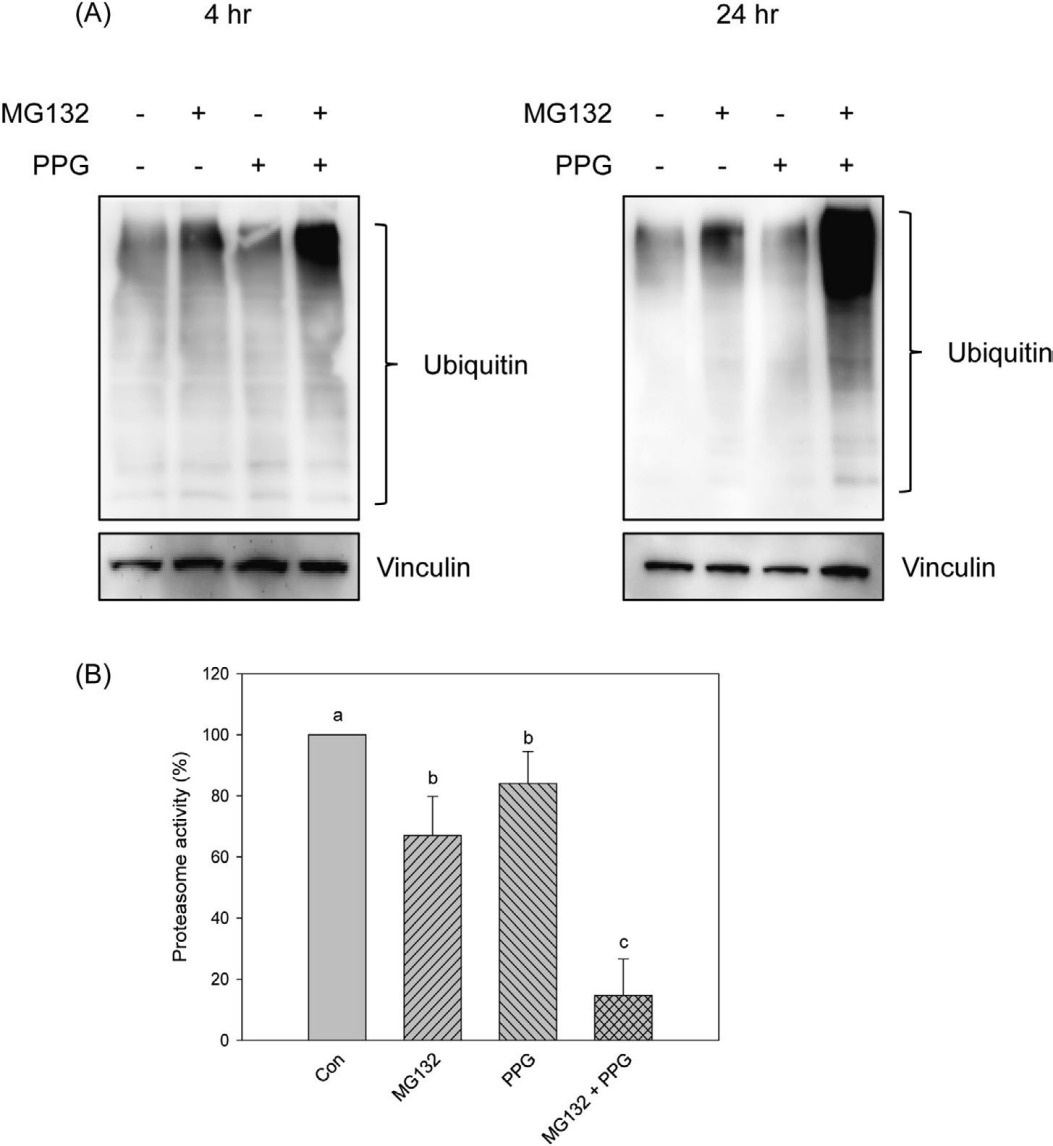
Suppression of proteasome activity by the combined treatment of MG132 and propolin G in MDA‐MB‐468 cells. MDA‐MB‐468 cells were subjected to treatment with 1 μM of MG132, 10 μM of propolin G (PPG) and the combination of MG132 (1 μM) and PPG (10 μM) for 24 h. The cell lysates were subsequently harvested, and (A) the expression levels of ubiquitin were assessed using western blot analysis, while (B) chymotrypsin‐like proteasome activity was measured utilizing the ELISA method. All experiments were conducted in triplicate, and results are presented as means ± SD. Mean values denoted with different subscript letters were found to be significantly different from those of the control group (*p* < 0.05).

The disruption of proteasome activity has been demonstrated to induce apoptosis in cancer cells (D'Arcy et al. 2015; Mir et al. 2023; Tian et al. 2014). To determine whether the induction of apoptosis by the combination treatment was mediated through a proteasome‐dependent mechanism, we evaluated the expression levels of ubiquitinated proteins and proteasome activity. A significant accumulation of ubiquitinated proteins was observed in cells subjected to the combination treatment for both 4 and 24 h (Figure 3A). Concurrently, proteasome activity was found to be inhibited in the groups treated with MG132, propolin G, and the combination treatment. Notably, the inhibitory effect was significantly more pronounced in the combination treatment group compared to those treated with MG132 or propolin G alone (Figure 3B).

### 3.4 Combination Treatment of Proteasome Inhibitor and Propolin G Induced Apoptosis Through the Activation of ER Stress

**FIGURE 4:**
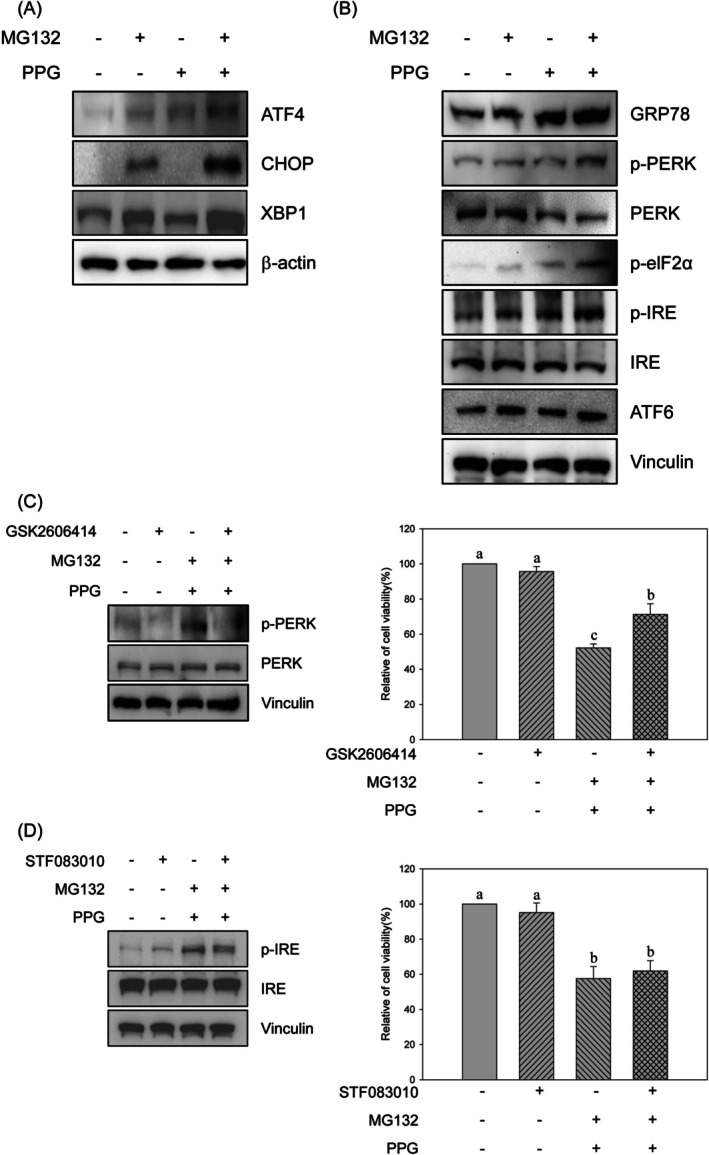
Induction of ER stress‐mediated cell death by the combined treatment of MG132 and propolin G in MDA‐MB‐468 cells. MDA‐MB‐468 cells were subjected to treatment with 1 μM of MG132, 10 μM of propolin G (PPG), and the combination of MG132 (1 μM) and PPG (10 μM) for 4 or 24 h. (A) Subsequently, the expression levels of genes regulated by UPR and (B) the upstream signals associated with the UPR were assessed using western blot analysis. (C) MDA‐MB‐468 cells were pre‐treated with a PERK inhibitor (GSK2606414, 10 μM) or (D) an IRE inhibitor (STF083010, 10 μM) prior to incubation with the combination of MG132 and PPG. Cell viability was subsequently evaluated using the MTT method. All experiments were conducted in triplicate, and the results are presented as means ± SD. Mean values denoted with different subscript letters were found to be significantly different from those of the control group (*p* < 0.05).

The inhibition of proteasome activity has been shown to induce ER stress through the activation of the UPR (Mimura et al. 2012; Obeng et al. 2006; Walter and Ron 2011). To further investigate whether the suppression of proteasome activity by the combination treatment leads to ER stress, the expression of UPR‐regulated genes was assessed. After 24 h of treatment, the expression levels of ATF4, CHOP, and XBP‐1 were significantly elevated in the combination treatment group compared with the groups treated with MG132 or propolin G alone (Figure 4A). Additionally, the upstream signals of the UPR were evaluated following 4 h of treatment. The expression of GRP78, a key regulator of the UPR in response to ER stress, was markedly upregulated in the combination treatment group. Moreover, the expression levels of p‐PERK, p‐elF2α, and p‐IRE were also significantly increased in the combination treatment group compared to the groups treated with MG132 or propolin G alone (Figure 4B). Although an induction of ATF6 expression was observed across all three treatment groups, no significant differences were noted between the combination treatment and the individual treatments with MG132 or propolin G. These results suggest that the induction of the UPR by the combination treatment may occur through p‐PERK‐ and p‐IRE‐mediated signaling pathways.

To further evaluate whether the cytotoxicity induced by the combination treatment was mediated through the activation of the UPR, specific inhibitors of PERK and IRE were investigated. The results indicated that the reduction in cell viability caused by the combination treatment was reversed following pretreatment with the PERK inhibitor GSK2606414 (Figure 4C). In contrast, pretreatment with the IRE inhibitor STF083010 did not restore the cell viability that was diminished by the combination treatment (Figure 4D). These findings suggest that the decrease in cell viability resulting from the combination treatment may be mediated through a PERK‐dependent signaling pathway.

### 3.5 Combination Treatment of Proteasome Inhibitor and Propolin G Induced Apoptosis Through the Autophagy‐Mediated Signaling Pathway

**FIGURE 5:**
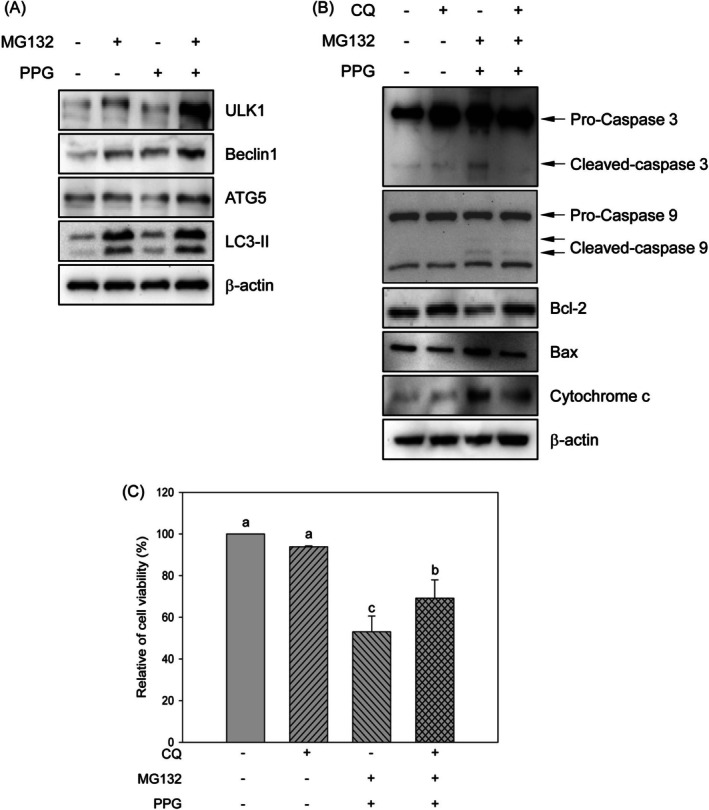
Activation of autophagy‐mediated apoptosis by the combined treatment of MG132 and propolin G in MDA‐MB‐468 cells. MDA‐MB‐468 cells were subjected to treatment with 1 μM of MG132, 10 μM of propolin G (PPG), and the combination of MG132 (1 μM) and PPG (10 μM) for 24 h. (A) The expression levels of autophagy markers were assessed using western blot analysis. Prior to incubation with the combination of MG132 and PPG, cells were pre‐treated with the autophagy inhibitor (chloroquine, CQ) at a concentration of 10 μM. (B) Cell lysates were subsequently collected to evaluate the expression of apoptotic regulators, while (C) cells were harvested to assess cell viability. All experiments were conducted in triplicate, and the results are presented as means ± SD. Mean values denoted with different subscript letters were found to be significantly different from those of the control group (*p* < 0.05).

The ubiquitin‐proteasome system and the autophagy‐lysosomal system are two major pathways responsible for protein degradation and maintaining proteostasis. Research has demonstrated that inhibiting proteasome activity can activate the autophagy‐lysosomal degradation pathway (Zhu et al. 2010). As illustrated in Figure 3, proteasome activity was significantly reduced in the group receiving combination treatment, highlighting the need for a more comprehensive investigation into the autophagy‐lysosomal signaling pathway. The expression levels of ULK1, Beclin1, ATG5, and LC3‐II were markedly increased in the combination treatment group (Figure 5A), indicating the activation of autophagy. Further analysis of the role of autophagy in apoptosis induced by the combination treatment revealed that the inhibition of autophagy through chloroquine pretreatment reversed the upregulation of apoptotic regulators—including cleaved caspase‐3, cleaved caspase‐9, Bax, and cytochrome c—induced by the combination treatment. Additionally, the decrease in Bcl‐2 protein levels resulting from the combination treatment was also restored following chloroquine pretreatment (Figure 5B). Notably, chloroquine pretreatment was found to restore cell viability that had been adversely affected by the combination treatment (Figures 5C).

## 4 Discussion

**FIGURE 6:**
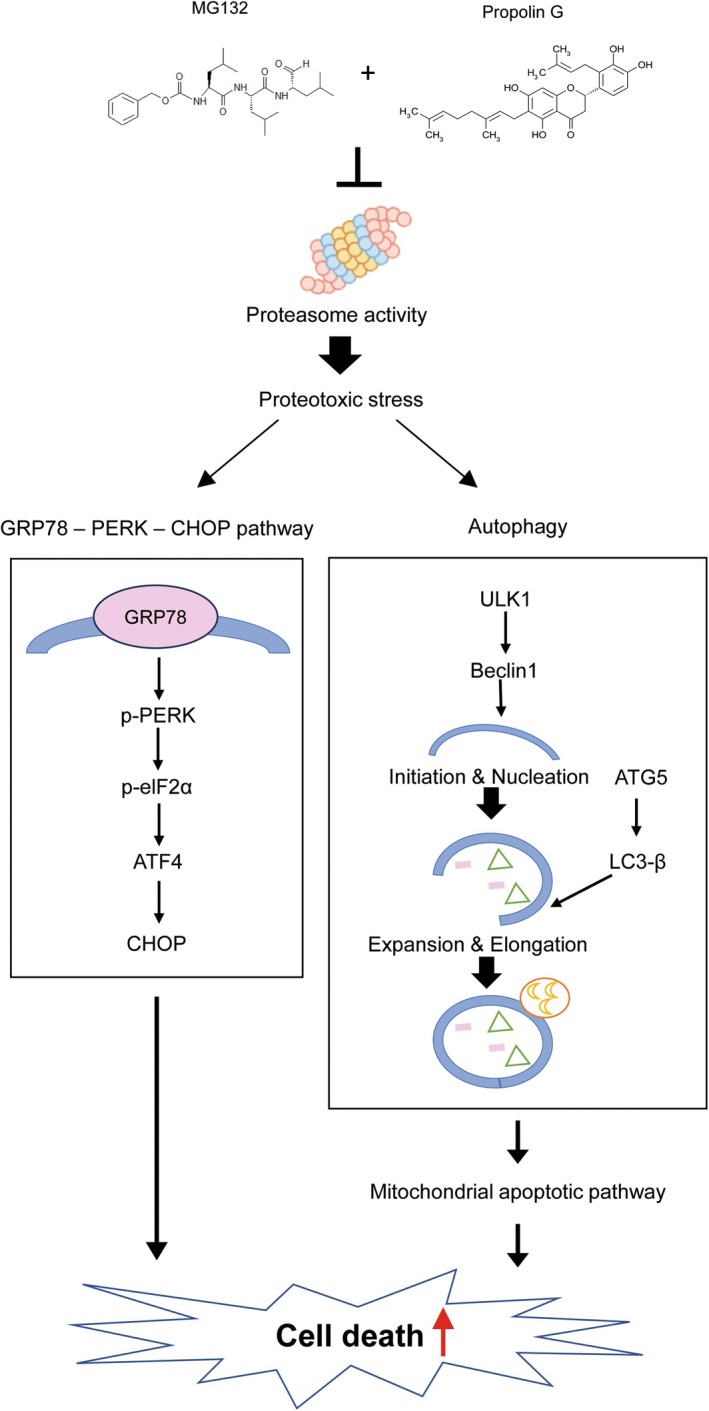
Schematic representation of the potential molecular mechanisms underlying the anticancer effects of the combined treatment of MG132 and propolin G in MDA‐MB‐468 cells.

Maintaining protein homeostasis, also known as proteostasis, is crucial for cellular function and survival. Disruption of proteostasis, which can occur due to impaired protein degradation, leads to the accumulation of damaged, immature, or misfolded proteins. This accumulation results in proteotoxic stress, which may ultimately trigger cell death (D'Arcy et al. 2015; Mir et al. 2023; Tian et al. 2014). Consequently, targeting protein degradation systems has emerged as a promising strategy for cancer treatment and prevention. In this study, we investigated the anticancer mechanisms associated with the combination of proteasome inhibitor MG132 and propolin G, a natural compound derived from Taiwanese propolis, in breast cancer cells. Our findings indicated that this combination significantly reduces cancer cell viability and induces apoptosis through mechanisms involving ER stress and autophagy (Figure 6).

The ubiquitin‐proteasome system (UPS) serves as a principal mechanism for protein degradation. Inhibition of the proteasome induces proteotoxic stress, which subsequently activates adaptive stress responses. Clinically, proteasome inhibitors, such as bortezomib, have demonstrated efficacy in the treatment of hematological malignancies; however, their utility in solid tumors remains limited (Moreau et al. 2012; Soave et al. 2017). In the context of breast cancer, there is evidence of elevated proteasome activity and overexpression of ubiquitin‐conjugating enzymes (Chen and Madura 2005). Notably, Basal‐like subtypes of breast cancer appear to exhibit increased sensitivity to proteasome inhibition (Petrocca et al. 2013). Consequently, targeting proteotoxic stress to induce cell death may represent a viable therapeutic strategy for breast cancer treatment. Nonetheless, the non‐selective cytotoxicity associated with proteasome inhibitors can adversely affect normal cells, resulting in side effects such as neuropathy and cardiovascular toxicity (Georgiopoulos et al. 2023; Soave et al. 2017). To mitigate these toxic effects while preserving therapeutic efficacy, combination strategies utilizing natural compounds have garnered significant interest. In this study, we employed minimally cytotoxic doses of MG132 and propolin G, which resulted in a synergistic reduction in cell viability, colony formation, and apoptosis.

Mechanistically, the inhibition of the proteasome by MG132 activates ER stress pathways through the accumulation of misfolded proteins. In our study, the combination treatment resulted in the upregulation of UPR markers, including ATF4, CHOP, and XBP1. It increased the p‐PERK and p‐IRE, alongside an elevation in GRP78 expression. Notably, the inhibition of PERK, but not IRE, restored cell viability, indicating a predominant role of the PERK signaling pathway in the induction of apoptosis. Additionally, autophagy was also activated, as evidenced by increased levels of ULK1, Beclin1, ATG5, and LC3‐II. The blockade of autophagy using chloroquine reversed apoptosis and restored Bcl‐2 levels and cell viability, suggesting that excessive autophagy may contribute to cell death. These findings suggest that ER stress and autophagy interact in response to proteasome inhibition, ultimately leading to apoptosis.

Natural compounds, such as propolin G, present several advantages, including low toxicity and the capacity to modulate multiple signaling pathways. Propolin G, in particular, has exhibited anticancer properties by inducing apoptosis, causing cell cycle arrest, and inhibiting EMT (Pai et al. 2022; Weng et al. 2007). Recently, propolin G has been identified as a selective inhibitor of histone deacetylase 6 (HDAC6) (Pai et al. 2022). HDAC6 plays a crucial role in regulating the aggresome‐autophagy pathway, which serves as a secondary clearance mechanism activated in response to proteasome impairment. This pathway mediates the transport of polyubiquitinated proteins along microtubules to aggresomes through interactions with dynein and α‐tubulin (Miki et al. 2011). The inhibition of HDAC6 disrupts this pathway, leading to unresolved proteotoxic stress and the activation of ER stress and autophagy (Chiu et al. 2019). Building upon these findings, we hypothesized that propolin G may enhance the efficacy of MG132 by interfering with HDAC6‐dependent aggresome formation. Our data indicated an increase in proteotoxic stress, activation of the UPR, and markers of autophagy under the combination treatment. Although direct evidence of HDAC6 inhibition by propolin G was not established in this study, prior reports lend support to this potential mechanism (Pai et al. 2022). It is noteworthy that both proteasome activity and autophagy are frequently elevated in cancer cells compared with normal cells, attributable to their heightened rate of protein synthesis, metabolism, and intrinsic stress levels (Galluzzi et al. 2015; Mathew et al. 2007). This increased reliance on proteostasis pathways may render cancer cells particularly susceptible to dual inhibition strategies. Such cancer‐specific dependence presents a potential therapeutic opportunity, enabling the combined inhibition of proteasome and autophagy pathways to target malignant cells while selectively preserving normal tissues. This context‐dependent vulnerability underscores the translational potential of this combination strategy in oncology. However, given the dual role of autophagy in cancer—where it can either promote survival or facilitate cell death depending on the context—further research is warranted to elucidate its precise contribution and optimize therapeutic selectivity.

In conclusion, our findings indicate that the combination of MG132 and propolin G synergistically induces apoptosis in breast cancer cells via ER stress and autophagy‐related mechanisms. This strategy, which is based on natural compounds, has the potential to enhance the efficacy of proteasome inhibitors while simultaneously reducing systemic toxicity. In addition to providing mechanistic insights, this combination therapy offers a promising translational approach. Future research should focus on determining optimal dosing schedules, the timing of treatment (whether sequential or concurrent), and delivery strategies, including nanoparticle formulations or localized administration, to enhance tumor selectivity and minimize off‐target effects. Moreover, investigating its compatibility with established therapies such as chemotherapy or radiotherapy may facilitate the positioning of this strategy as a viable treatment option for tumors that are dependent on proteostasis or exhibit drug resistance. These findings offer a conceptual framework for the development of targeted combination therapies that leverage cancer‐specific vulnerabilities in protein homeostasis.

## Author Contributions

**Jih‐Tung Pai:** conceptualization (equal), methodology (equal), investigation (equal), and writing – original draft (equal). **Lei‐Po Chen:** methodology (equal), investigation (equal), data curation (equal), visualization (equal), and writing – original draft (equal). **Hsuan‐Jui Chang:** investigation (supporting), validation (supporting), and visualization (supporting). **Shih‐Wei Wang:** formal analysis (supporting) and validation (supporting). **Yann‐Lii Leu:** resources (equal) and supervision (supporting). **Cheng‐Ta Lai:** writing – review and editing (equal), supervision (equal), and project administration (equal). **Meng‐Shih Weng:** conceptualization (equal), funding acquisition (equal), supervision (equal), and writing – review and editing (equal).

## Ethics Statement

The authors have nothing to report.

## Conflicts of Interest

The authors declare no conflicts of interest.

## Data Availability

The datasets generated or analyzed in this study are available from the corresponding author upon reasonable request.
